# Flipping the script: Advances in understanding how and why P4-ATPases flip lipid across membranes

**DOI:** 10.1016/j.bbamcr.2024.119700

**Published:** 2024-02-19

**Authors:** Adriana C. Norris, Alexander J. Mansueto, Mariana Jimenez, Eugenia M. Yazlovitskaya, Bhawik K. Jain, Todd R. Graham

**Affiliations:** Department of Biological Sciences, Vanderbilt University, Nashville, TN, USA

**Keywords:** Flippase, Membrane asymmetry, Vesicular transport, Phosphatidylserine, Glucosylceramide, P-type ATPase

## Abstract

Type IV P-type ATPases (P4-ATPases) are a family of transmembrane enzymes that translocate lipid substrates from the outer to the inner leaflet of biological membranes and thus create an asymmetrical distribution of lipids within membranes. On the cellular level, this asymmetry is essential for maintaining the integrity and functionality of biological membranes, creating platforms for signaling events and facilitating vesicular trafficking. On the organismal level, this asymmetry has been shown to be important in maintaining blood homeostasis, liver metabolism, neural development, and the immune response. Indeed, dysregulation of P4-ATPases has been linked to several diseases; including anemia, cholestasis, neurological disease, and several cancers. This review will discuss the evolutionary transition of P4-ATPases from cation pumps to lipid flippases, the new lipid substrates that have been discovered, the significant advances that have been achieved in recent years regarding the structural mechanisms underlying the recognition and flipping of specific lipids across biological membranes, and the consequences of P4-ATPase dysfunction on cellular and physiological functions. Additionally, we emphasize the requirement for additional research to comprehensively understand the involvement of flippases in cellular physiology and disease and to explore their potential as targets for therapeutics in treating a variety of illnesses. The discussion in this review will primarily focus on the budding yeast, *C. elegans*, and mammalian P4-ATPases.

## 1. Introduction

Biological membranes are crucial to all living cells and substantial cellular resources are used to produce, maintain and remodel membranes as cells divide and respond to changing environmental conditions. This is especially true for eukaryotic cells where intracellular, organellar membranes can constitute as much as 98 % of the total membrane [1–3]. For a typical cellular membrane, about half of the mass is protein and half lipid, and these two components are highly interdependent in producing the appropriate fluidity and selective permeability of the bilayered structure [1,4]. The plasma membrane and each membrane-bound organelle have their own unique composition of lipids, which strongly modulate the function of proteins embedded in the membrane [5,6]. Thus, membrane-centric processes like the exchange of stored potential energy in ATP for that in ion gradients, transport of nutrients and waste in and out of cells and organelles, vesicular transport, cytokinesis, cell fusion, signal transduction, and propagation of action potentials, are all remarkably dependent on the finely-tuned composition of membranes. In fact, each leaflet of a single bilayer can have very different lipid compositions, yielding a membrane with transverse asymmetry between the two surfaces [7–12].

**Fig. 1.**
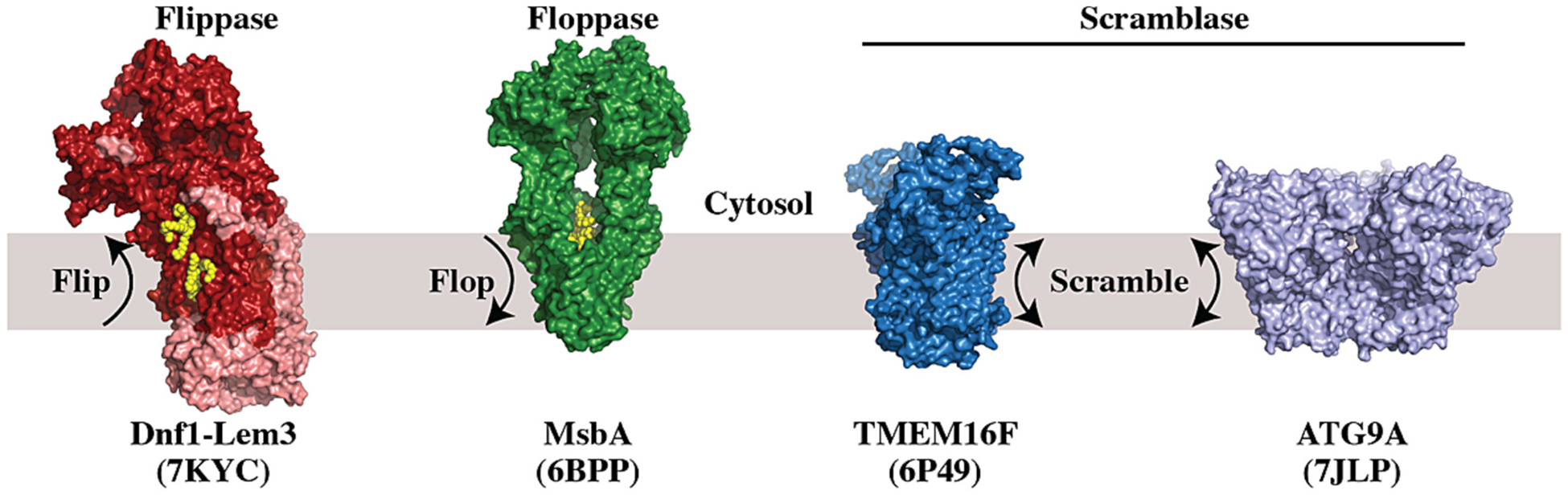
Membrane asymmetry is controlled by flippases, floppases and scramblases. A few examples of lipid transporter structures with Protein Data Bank Identifiers. Substrate lipid is shown in yellow for Dnf1-Lem3 and MsbA.

Membrane asymmetry is controlled by a set of lipid transporters called flippases, floppases, and scramblases (Fig. 1) [7,13]. Lipid fl*i*ppases mediate the ATP-powered *i*nward transport of lipid from the outer leaflet of the plasma membrane to the cytosolic leaflet and this activity is primarily catalyzed by type IV P-type ATPases (P4-ATPases) [14,15]. Classical substrates of the P4-ATPases include phosphatidylserine (PS) and phosphatidylethanolamine (PE) and these lipids are highly enriched in the cytosolic leaflet of the plasma membrane and are nearly absent in the extracellular leaflet [9,10,12,16–18]. Lipid fl*o*ppases, primarily ABC transporters, catalyze the *o*utward transport of lipid. Classical lipid substrates of ABC transporters include lipid A in bacterial cells transported by MsbA [19], and phosphatidylcholine (PC) and cholesterol in animal cells [20–23]. Although the mammalian ABC transporters have well-established roles in exporting lipids to extracellular destinations such as bile or high-density lipoprotein (HDL), their role in controlling membrane asymmetry in most eukaryotic cells is not as well characterized. Finally, scramblases catalyze energy-independent and bidirectional lipid transport. Scramblases fall into several different protein families, do not exhibit any lipid specificity, and play important roles in membrane biogenesis and the regulated disruption of membrane asymmetry at the plasma membrane [24–31].

Spatial and temporal regulation of lipid transporters enables them to influence membrane organization at various places and times within the cell. Membrane biogenesis starts at the endoplasmic reticulum (ER) and most of the lipid biosynthetic reactions occur in the cytosolic leaflet; therefore, the rapid movement of lipids between leaflets is needed for the balanced growth of the membrane [32,33]. In contrast to artificial membranes where phospholipid flip-flop between leaflets is exceptionally slow, the ER membrane exhibits rapid and energy-independent phospholipid flip-flop, which may be mediated by phospholipid scramblases [33]. Recently, two mammalian ER-resident proteins that have scramblase activity in vitro, VMP1 and TMEM41B, have been identified [24,25,29,34]. These proteins are important for autophagosome formation, β-coronavirus replication, lipid droplet formation, and lipoprotein secretion, however, the influence of VMP1 and TMEM41B on overall membrane biogenesis in the ER remains unclear (32–35).

As the membrane moves from the ER through the Golgi complex to the plasma membrane, P4-ATPases localized to Golgi cisternae and the plasma membrane flip PS and PE to the cytosolic leaflet in order to generate the membrane asymmetry characteristic of the plasma membrane [35–39]. Many of the P4-ATPases continually traffic between the plasma membrane, endosomes, and Golgi complex, so membrane asymmetry is also maintained within the endosomal system [39–44]. This gradient of phospholipid within the membrane can serve as a signal transduction platform; similar to how ion gradients established by P-type ATPases are used. For ion gradients, the regulated opening of ion channels transduces the signal while for lipid gradients in the plasma membrane, it is the activation of scramblases, such as TMEM16F or Xkr8, and exposure of cytosolic leaflet lipids in the exofacial leaflet [13]. During apoptosis, the caspase-dependent activation of Xkr8 and inactivation of P4-ATPases breaks the asymmetry and exposes PS and PE in the extracellular leaflet. Exposed PS is a prominent “eat me” signal, which is recognized by adjacent cells or macrophages to stimulate the phagocytosis of cell corpses [31,45–48]. Regulated exposure of inner-leaflet lipids also contributes significantly to blood clotting reactions, cytokinesis, and cell-cell fusion events [30,49–53]. Thus, the making and breaking of membrane asymmetry are crucial for tissue homeostasis.

In addition to their prominent role in establishing membrane asymmetry, lipid transport by P4-ATPases is tightly linked to membrane remodeling events required for vesicle-mediated transport, polarized cell growth, regulation of organellar pH and fusion, and extracellular vesicle production [35–37,41–43,54–64]. There are 14 different human P4-ATPase genes and mutations in these genes are linked to a broad spectrum of diseases (described below). In addition, P4-ATPases are required for virulence by fungal pathogens, such as *Candida albicans* and *Cryptococcus neoformans* [55,65–68]. The P4-ATPases were once thought to only transport PS and PE, but we now know that certain P4-ATPases flip phosphatidylcholine (PC), glucosylceramide (GlcCer) and galactosylceramide (Table 1) [69–73]. In this review, we will describe the substantial progress that has been made in recent years to understand the structural basis for how the P4-ATPases recognize and flip specific lipids across membranes, how new lipid substrates have been identified, regulatory mechanisms that control flippase activity, insights into how P4-ATPases evolved from primordial cation pumps, and the cellular and physiological consequences of P4-ATPase dysfunction. We will focus our discussion on the budding yeast, *C. elegans*, and mammalian P4-ATPases and refer readers to excellent recent reviews on the plant P4-ATPases (ALA1- ALA12 in *Arabidopsis*) [74,75].

## 2. Atypical P4-ATPase substrates

P4-ATPases have a much broader set of substrate lipids than originally anticipated. The initial studies of the aminophospholipid translocase of erythrocyte membranes found that the transport activity was specific for spin-labeled lipid derivatives of PS and PE, while PC derivatives were transported slowly or not at all [16,17,76]. In contrast, a lipid flippase activity for PC and PE, but not PS, was identified in the budding yeast plasma membrane [72,73]. We now know that PS/PE transport in erythrocytes is primarily catalyzed by ATP11C-CDC50A and PC/PE transport across the yeast plasma membrane is catalyzed by Dnf1-Lem3 and Dnf2-Lem3 [69,77]. Budding yeast localizes their PS/PE transporters (Drs2, Neo1 and Dnf3) primarily to the Golgi complex and endosomal system [35–37]. Because the membrane constantly fluxes between the Golgi, plasma membrane, and endosomes by vesicular transport, the internally localized PS/PE flippases can maintain PS and PE asymmetry of the plasma membrane. Additionally, in budding yeast, the observation that most of the plasma membrane PC is confined to the cytosolic leaflet while glycosphingolipids are mainly confined to the extracellular leaflet is likely due to the activity of PC/PE flippases (Dnf1 and Dnf2) [78]. Dnf1 and Dnf2 may actually prefer lysophospholipid substrates bearing a single acyl chain (LysoPC or LysoPE) to diacylated species [79,80]. Mammals also express PC flippases (ATP10A, ATP10B, ATP8B2) [81,82] but the cell types that endogenously express these P4-ATPases are not well defined, nor has their influence on membrane asymmetry been determined. It is possible that the current views of membrane organization where PS/PE occupies the cytosolic leaflet and PC is enriched in the extracellular leaflet may not apply to all cell types. The biological significance of PC transport by P4-ATPases is still an open question.

GlcCer is a newly discovered, highly conserved, and surprising substrate of the Dnf1/2-ATP10A/B/D clade of P4-ATPases [70,82]. GlcCer is built on a sphingosine backbone instead of glycerol, it lacks a phosphate group, and it has a glucose molecule for its headgroup. The discovery that Dnf1/2 flips GlcCer came from a study addressing how these proteins distinguish PC from sphingomyelin, a sphingolipid that has the exact same phosphocholine headgroup as PC but is not a substrate for these flippases [83]. Our group was able to evolve variants of Dnf1 that substantially enhanced sphingomyelin transport. However, when examining other sphingolipids to assess the specificity of the mutants, we were surprised to find that the wild-type Dnf1 and Dnf2 robustly transport GlcCer. Dnf1/2 orthologs from widely divergent fungi, such as *Candida albicans* and *Schizosaccharomyces* pombe, as well as the human ATP10A, ATP10B, and ATP10D, all transport GlcCer [55,70,82]. The reason the translocation of this substrate is so highly conserved remains unknown. However, it is noteworthy that mutations that specifically impair GlcCer transport via the *Candida albicans* Dnf2 abrogate polarized hyphal growth and infectivity of this opportunistic pathogen [55].

ATP8B1 has been reported to be a cardiolipin transporter because the level of its expression correlates with the degree of fluorescently-tagged cardiolipin uptake in alveolar cells [84]. However, cardiolipin does not stimulate the ATPase activity of purified ATP8B1 [85], suggesting that ATP8B1 does not directly transport this lipid. The precise substrate specificity of ATP8B1 has been controversial [81,85–89], with ectopic expression studies attributing PS transport activity [88,89] or PC transport activity to ATP8B1 [81]. The purified and reconstituted ATP8B1 displayed primarily a PS transport activity with a much weaker activity towards PC [86]. Thus, it is possible that the membrane environment, posttranslational modification of ATP8B1, or regulatory interactions can modulate the substrate specificity of this transporter. Additionally, ATP8B2 has been shown to translocate plasmalogens, alkenyl-ether glycerophospholipids harboring a vinyl ether linkage at the sn-1 position and typically an ethanolamine headgroup [90]. Translocation of plasmalogen from the extracellular leaflet to the cytosolic leaflet presents this lipid to an ill-defined regulatory system that leads to the degradation of Far1, the rate-limiting enzyme acting in plasmalogen synthesis. Thus, by flipping this lipid to the cytosolic leaflet, ATP8B2 serves an important role in plasmalogen homeostasis.

## 3. P4-ATPase structure

**Fig. 2.**
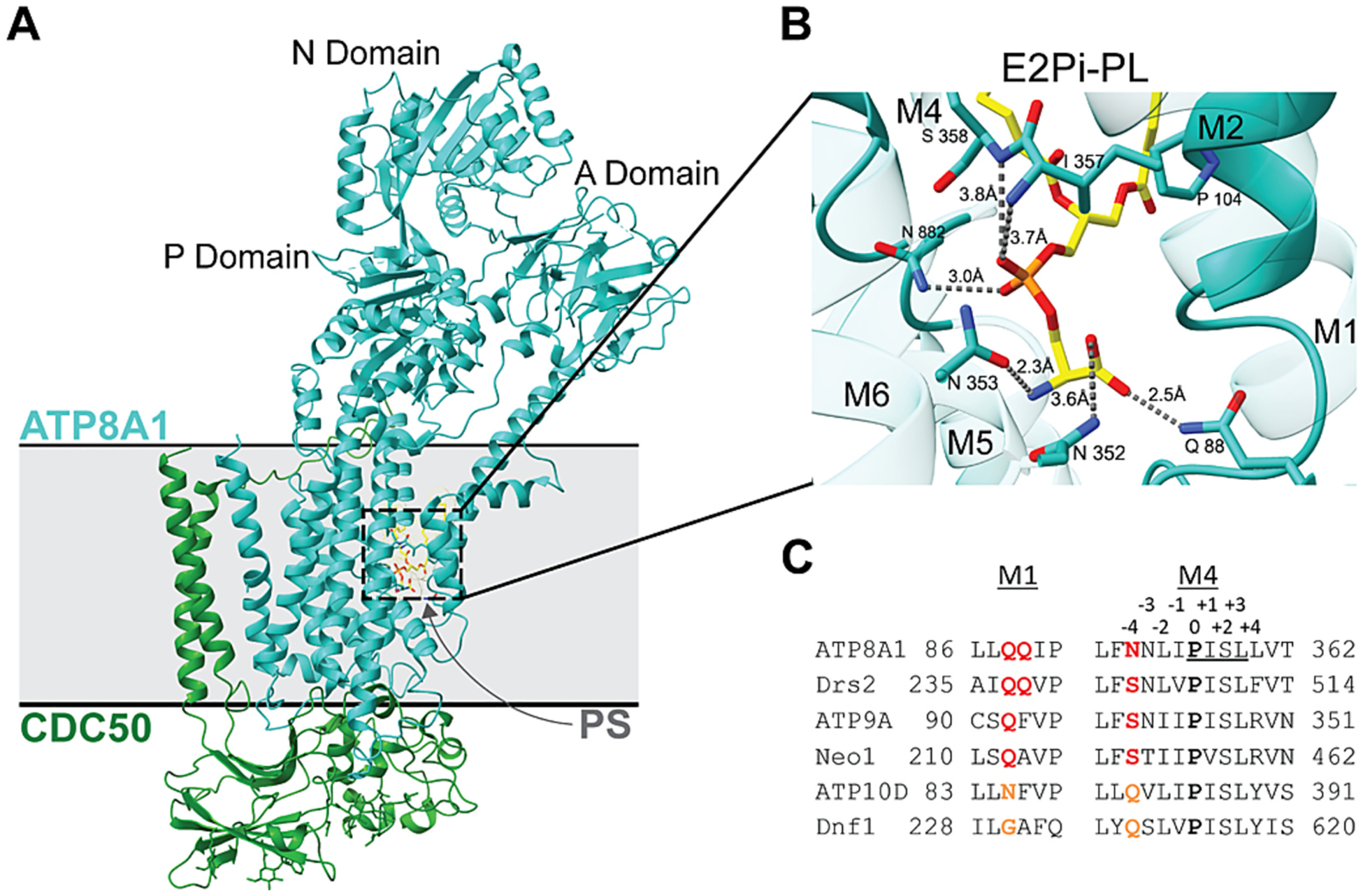
Structure of ATP8A1-CDC50 in the E2P state bound to phosphatidylserine (PS). (A) Structure of ATP8A1-CDC50 with PS occluded in the entry gate (PDB: 6K7M). The P, N, and A domains are highlighted. The phospholipid binding site view in (B), depicts key residues in M1, M4, and M6 interacting with the phospholipid head group. αTM6 is shown in the foreground and M2 in the background. Hydrogen bond interactions are shown as dashed lines with corresponding distances labeled. (C) Alignment of M1 and M4 sequences that help form the lipid binding site. The position of residues relative to the highly conserved proline (at position 0 in M4) is indicated by the −4 to +4 values.

Multiple P4-ATPase cryogenic electron microscopy (cryoEM) structures in nearly all conformational states of the catalytic cycle have appeared in the literature over the past few years, including ATP8A1-CDC50A, ATP8B1-CDC50A, ATP11C-CDC50A, Drs2-Cdc50, Dnf1-Lem3, Dnf2-Lem3, and Neo1 [86,91–97]. Most P4-ATPases are αβ heterodimers comprised of the catalytic α subunit from the P4-ATPase gene family and a noncatalytic β subunit from the CDC50/LEM3/TMEM30 gene family (Table 1) [88,98–101]. The exception is the ATP9A/ATP9B/NEO1/TAT-5 group, which function without a β subunit [95,102]. There are three isoforms of the beta subunit in mammals, CDC50A/TMEM30A, CDC50B/TMEM30B, and CDC50C/TMEM30C, where CDC50A/TMEM30A is the most abundantly expressed isoform and interacts with most of the P4-ATPases [99,103]. The structural features are highly conserved between the mammalian and yeast P4-ATPases. The catalytic α subunits have a 10-transmembrane (TM) segment membrane domain, a large cytosolic loop between TM4 and TM5 that forms the nucleotide-binding N domain and phosphorylation (P) domain, and an actuator (A) domain formed from the cytosolic loop between TM2 and TM3 and part of the cytosolic N-terminal tail (Fig. 2). The remainder of the N-terminus and the cytosolic C-terminal tail can act as regulatory domains that serve to autoinhibit flippase activity by binding to the catalytic N, P, and A domains [85,86,94,104,105]. The α subunit extracellular loops tend to be short; however, in heterodimeric P4-ATPases, the second extracellular loop between TM3 and TM4 extends out from the membrane and is embedded in the large, glycosylated extracellular domain of the β subunit.

The β subunit has two TM segments that pack against αTM10 and its extracellular domain snugly caps the entire membrane domain of the α subunit [91,92,94,96,97](Fig. 2). Extensive contacts between the extracellular loops of α and the extracellular domain of β help hold αTM3–10 in a rigid conformational state, leaving αTM1 and αTM2 more mobile during the pumping cycle. The cytosolic, N-terminal tail of β is partially resolved in available structures and makes a conserved interaction with a loop that extends from the αTM4 helix to the P domain [92,94,96,97]. In Dnf1-Lem3, the β N-terminus extends all the way to the A domain where a second site of interaction can be seen [96]. The β C-terminal tail is also cytosolic and runs along the C-terminal tail of the α subunit. The αβ subunits are co-dependent for their folding and exit from the ER [98,106]. In addition, the β subunit can contribute to substrate selection and likely participates in the regulation of the pump activity [96,106].

**Fig. 3.**
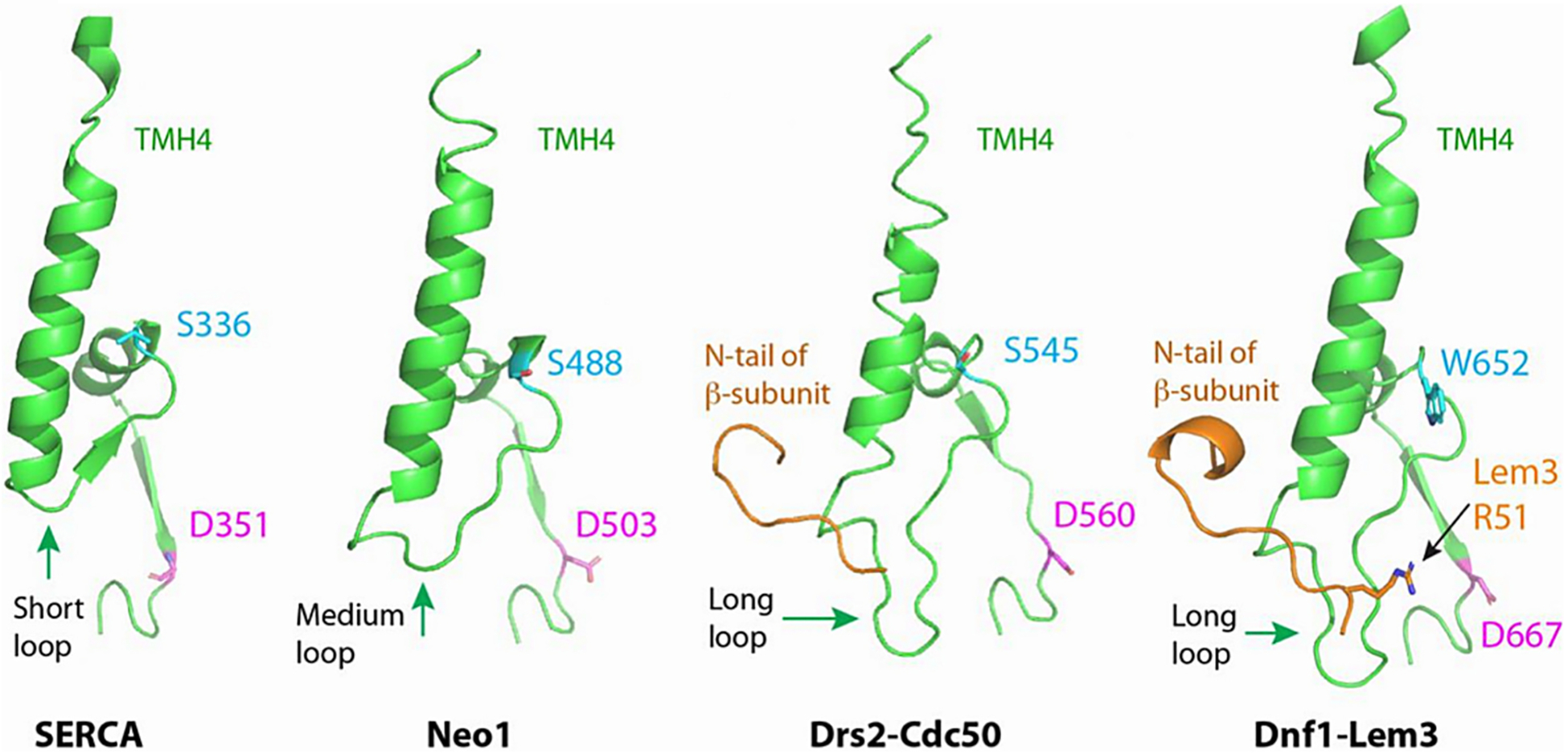
Extension of the cytosolic loop connecting TM4 to the P-domain in P4 ATPases relative to SERCA (P2-ATPase). A portion of each TM helix 4 (TMH4) is shown and the cytosolic extension of this helix is below the membrane border. The loop connecting TMH4 to the P domain in the monomeric Neo1 is intermediate in length between SERCA and the heterodimeric P4-ATPases. The functionally essential Neo1-S488 and Dnf1-W652 exit-gate residues (cyan) are positioned at the beginning of the P domain first helix and seem to form a substrate backstop in the exit gate that is closely linked to the phosphorylated aspartate (magenta residue). The N-terminal tail of the β-subunit (orange) associates with this loop and in the case of Dnf1 helps position Lem3-R51 for substrate interaction. Adapted from reference 90.

The Neo1 structure is especially informative because this monomeric P4-ATPase may represent an ancestral intermediate in P4-ATPase evolution from cation transporters [95,107]. The overall architecture of Neo1 is nearly identical to the α subunits of heterodimeric P4-ATPases [95], however, there are a few key differences. For example, the extracellular loops in Neo1 are very short and similar in length to the same loops in the Ca^++^-ATPase. This suggests that the longer loops in dimeric α subunits, particularly between TM2 and TM3, evolved in order to help mediate the β subunit interaction. A second interesting difference is that the loop connecting TM4 to the P domain in Neo1 is intermediate in length between SERCA and the dimeric α subunits (Fig. 3). Within the heterodimeric α subunits, this longer loop binds to the β N-terminus and positions it along the substrate translocation pathway [96]. Thus, the elongation of the loop connecting TM4 to the P domain may have been an important process in P4-ATPase evolution.

## 4. P4-ATPase transport mechanism

**Fig. 4.**
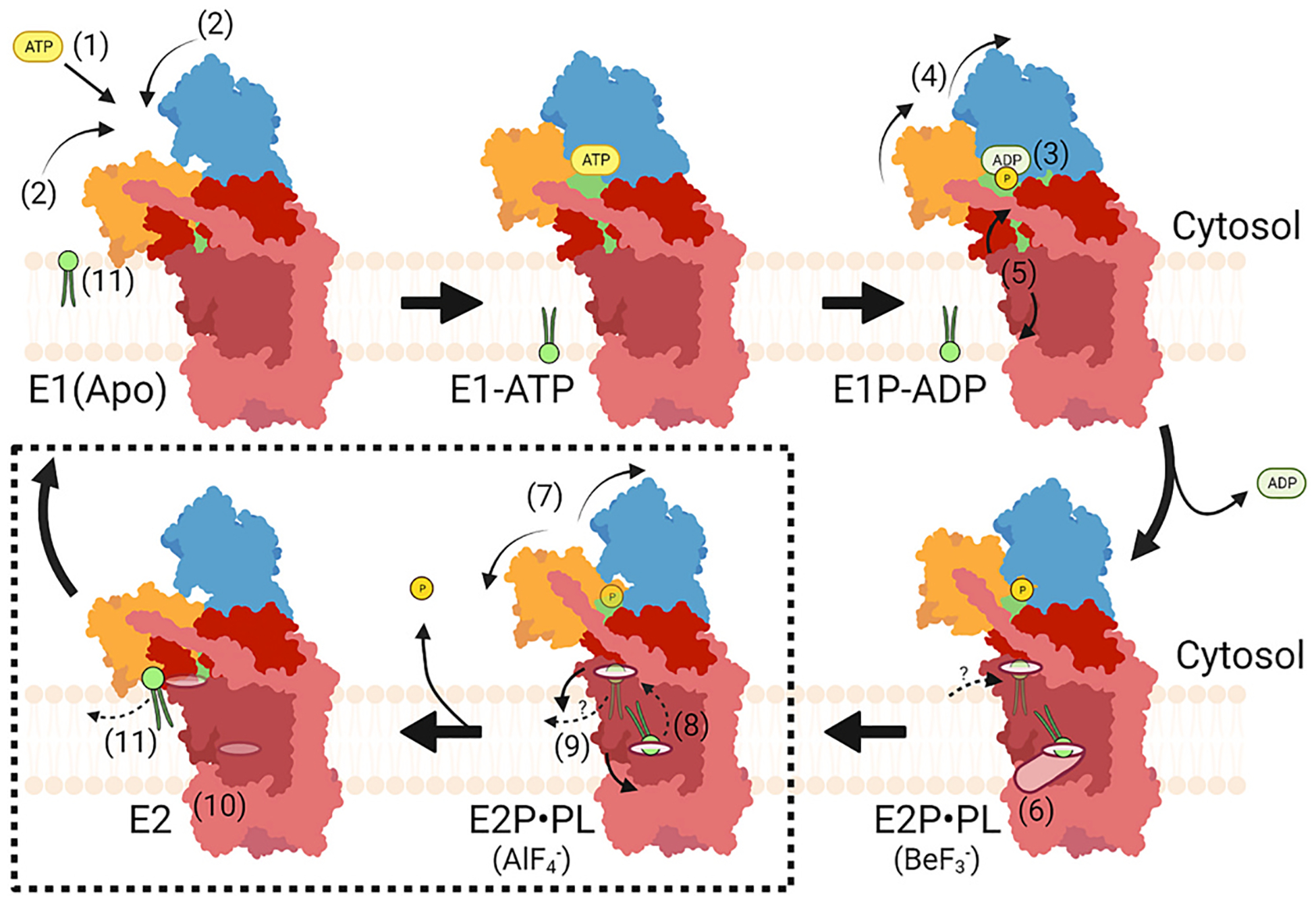
Model of the Post-Albers cycle for phospholipid transport by Dnf1/2-Lem3. The movement of cytosolic domains and lipid substrate during the Post-Albers cycle for Dnf1/2-Lem3 based on surface renditions. (1) ATP binds the N domain of E1 (Apo) relaxed state (7KY6). (2) A and N domains move towards each other. (3) The ATP γ-phosphate is transferred to an aspartate in the P domain. (4) A and N domains move clockwise to release ADP. (5) TM1 and TM2 tilting movements form the entry and exit gate binding sites (shown as halos). (6) Phospholipid binds to the entry and exit gate region of the E2P $\left({\text{BeF}}_{3}^{-}\right)$ conformation (7KYC) with the entry gate open to the exoplasmic leaflet. The source of the lipid in the exit gate at this stage is unclear but may derive from the cytosolic leaflet (dotted arrow). The dotted box encloses presumed structural states for which experimentally determined structures are lacking for Dnf1/2-Lem3. Structures of PS flippases stabilized with ${\text{AlF}}_{4}^{-}$ have substrate occluded in the entry gate. (7) The N and A domains become more flexible as the aspartyl-phosphate is hydrolyzed and Pi is released. (8) Phospholipid substrate flips to the cytosolic side at some point in the E2P to E2 steps.(9–10) TM1 and TM2 tilts back, closing the entry gate and widening the exit gate. (11) Phospholipid is ejected from the flippase. Domain coloring: Yellow – actuator (A) domain, Blue – nucleotide-binding (N) domain, Green – phosphorylation (P) domain, Muted red shade – transmembrane domain, Bright red shade – linkers from transmembrane segments to A, N, and P Domains, Salmon – Lem3.

**Fig. 5.**
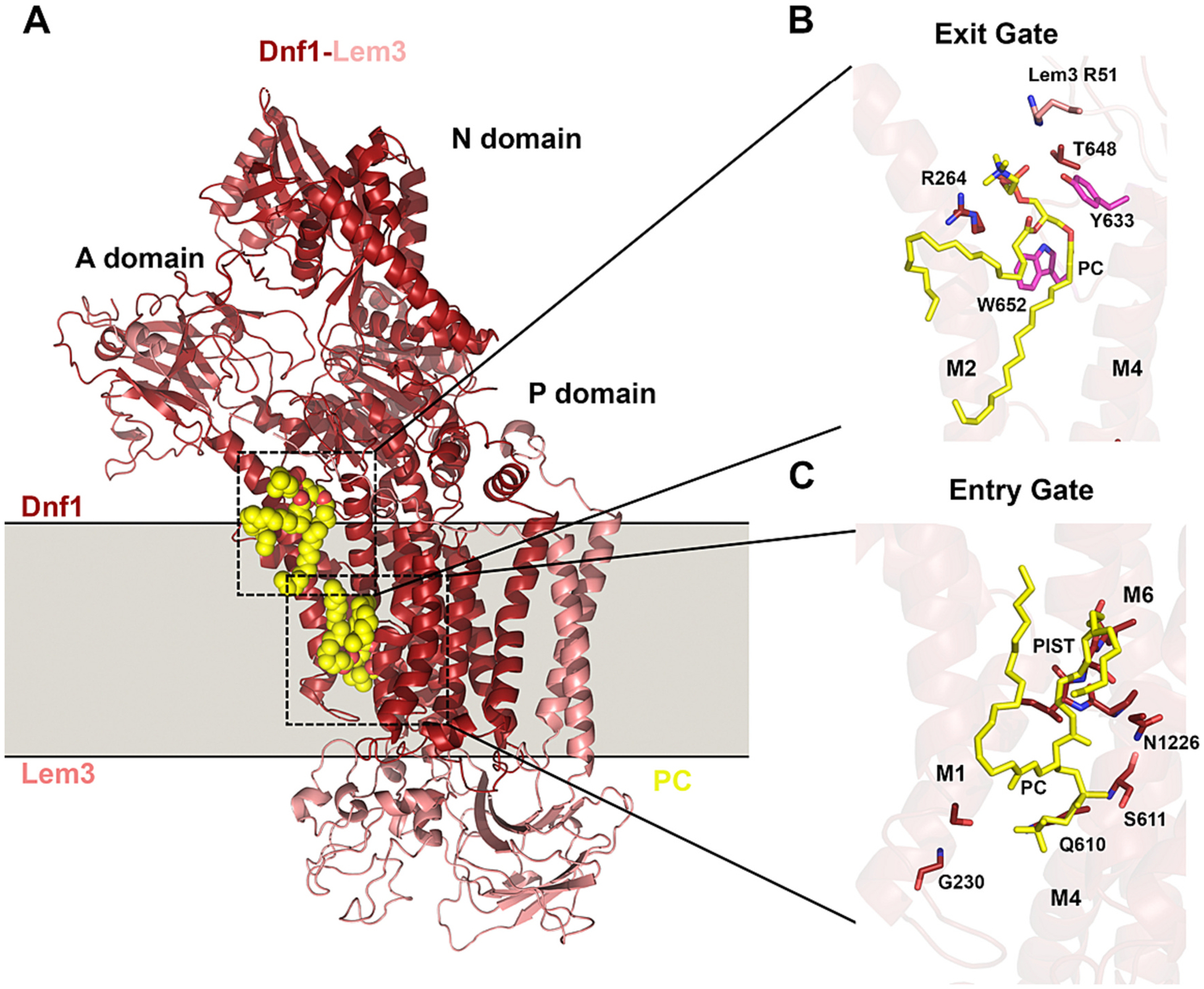
Structure of Dnf1-Lem3 with lipid bound. (A) Structure of the Dnf1-Lem3 in E2P state with phosphatidylcholine (PC) at the entry gate and exit gate (PDB: 7KYC). The major domains are highlighted. A close-up view of the proposed exit gate binding site (B) and entry gate binding site (C) is shown.

P4-ATPases use a modified version of the Post-Albers cycle to couple ATP hydrolysis to substrate transport across the membrane [108–110] (Fig. 4). This 4-stage cycle starts with the flippase in an E1 conformation that binds ATP in the N domain to form the E1•ATP conformational intermediate. The enzyme then transfers the γ-phosphate to an aspartic acid within the P domain DKTGT motif to transiently form the E1~P•ADP intermediate. Unlike cation transporters, no known substrate is required for this autophosphorylation event or is transported across the membrane during the E1 to E1~P transition [93,110]. In addition, no significant conformational changes within the membrane domain or β-subunit is associated with ATP binding and autophosphorylation (E1→E1•ATP→E1~P•ADP) and only slight movements are observed for the A and N domains [92,93,96]. The uncoupling of phosphorylation from membrane domain movements and transport events is a substantial difference from other P-type ATPases and represents an important event in the evolution of P4-ATPases [93]. Concomitant with ADP egress from the N domain, movement of the A domain and TM1,2 into the E2~P state allows substrate loading from the extracellular side of the membrane domain through an entry gate and binding to a site formed by residues in TM1, 2, 4, and 6 (E2~P•PL). The presence of substrate in this entry gate binding site appears in the E2~P intermediate stabilized with ${\text{BeF}}_{3}^{-}$ or ${\text{AlF}}_{4}^{-}$ with the A domain DGET motif poised to hydrolyze the aspartyl-phosphate [91–93]. The intermediates stabilized by ${\text{BeF}}_{3}^{-}$ typically maintain an open entry gate pathway from the cytosolic leaflet to the substrate binding site [91,94,96]. A subtle movement of TM1,2 closes off access to the cytosolic leaflet and occludes the lipid headgroup in several structures of PS flippases stabilized by ${\text{AlF}}_{4}^{-}$ [91–93]. Several structures with a substrate in the entry gate binding site are in this E2~P•PL conformation while none of the E1 structures have a substrate in the entry gate. The substrate lipid then flips as it moves past a region described as the hydrophobic gate and appears to dock at a second position called the exit gate binding site [95,96,111](Figs. 4 and 5). At some point in this substrate-flipping pathway, the actuator domain completes the dephosphorylation reaction, inducing the final E2~P→E2→E1 transition. Tilting motions of TM1–2 during the dephosphorylation reaction close the entry gate and widen the exit gate, events that presumably induce flipping and ejection of the substrate into the cytosolic leaflet. However, structures for transport states with the lipid captured in the process of flipping (dotted box in Fig. 4) are still missing and the precise translocation path is unknown.

### 4.1. Substrate translocation pathway-entry gate binding site

The substrate translocation pathway was initially characterized through mutagenic and functional studies [70,79,83,109–113], then subsequently defined and extended by cryoEM structures bearing lipid in the entry and exit gate sites [91–93,96,97]. The mutagenic studies included TM segment-swapping strategies between yeast P4-ATPases with different substrate specificities (Drs2 and Dnf1), which indicated TM1–4 contained critical residues for substrate discrimination [70,79,111,112]. Finer mapping of the individual residues identified key positions required for PS and/or GlcCer transport. Residues determining substrate specificity that clustered near the exoplasmic side of the membrane were proposed to form an entry gate and those closer to the cytosolic leaflet were described as exit gate residues. Within the entry gate, a Gln residue in the extracellular half of TM1 is highly conserved in PS flippases and is critical for PS transport and establishment of PS asymmetry (Fig. 2) [56,111]. The Gln in this position also appears to prevent GlcCer transport [70]. For example, the Dnf1 and Dnf2 GlcCer/PC flippases have a Gly in this TM1 position (G230 and G268, respectively) but the substitution of Gly for Gln converts these flippases to PS/PC flippases. The reciprocal mutation in Drs2 (Gln237Gly Gln238Ala) disrupts PS recognition without noticeably perturbing PE transport [111]. Similarly, the Q209G mutation in Neo1 disrupts PS recognition without perturbing PE transport [95,114]. Our group has introduced thousands of mutations in the membrane segments of yeast P4-ATPases and the TM1 Gln has the strongest and most specific impact on PS transport we have observed.

The first report of a P4-ATPase structure bearing substrate in the entry gate validated the findings from our mutation studies, as it revealed the entry gate TM1 Gln of ATP8A1 (Q88) forming a hydrogen bond between the side chain amine of Gln and the PS headgroup carboxyl (Fig. 2) [92,93]; it is the presence of this carboxyl group that distinguishes PS from PE (decarboxylation of PS produces PE). Thus, the structural and mutagenesis approaches converged on a residue crucial for PS recognition at the entry gate. Strikingly, a clinical study has also highlighted the importance of this residue in human P4-ATPases [115]. A de novo Q84E mutation in TM1 of ATP11A was associated with neurological deterioration in a patient and in a heterozygous mouse model. ATP11A-Q84E did not significantly disrupt PS transport but instead caused a striking increase in PC transport. Thus, the dominant neurological phenotype of ATP11A-Q84E is likely caused by inappropriate transport of PC and the depletion of PC from the outer leaflet of the plasma membrane. This same mutation in ATP8A2 (Q107E) or ATP11C (Q79E) significantly attenuated PS transport while conferring PC transport on ATP11A but not ATP8A2 [115]. Therefore, residues surrounding the lipid headgroup in the entry gate also have a substantial influence on what lipid is selected for transport.

Residues within TM4 and TM6 are also critical for substrate binding in the entry gate [70,91,92,95,96,112–114](Fig. 2, Fig. 5). Within almost all P-type ATPases, TM4 has a proline residue near the middle of the segment that breaks the α helix and frees up backbone C=O and N–H groups for substrate interactions. Most P4-ATPases have a conserved PISL motif in this position where backbone N–H groups engage the PS phosphate group (Fig. 2B, I357). Another key determinant of substrate specificity lies in the Pro – 4 (proline minus 4) position in TM4 where a Gln is essential for GlcCer transport by yeast Dnf1 and Dnf2, and mammalian ATP10A and ATP10D (Fig. 2B.C) [70]. The PS transporters have either an Asn or Ser in this position and prior mutagenesis studies highlighted the importance of the Pro – 4 Asn for PS recognition and transport by ATP8A2 [109]. In ATP8A1 and Drs2, both the Pro – 3 and Pro – 4 side chains engage in hydrogen bonds with the PS headgroup [92,93]. A highly conserved Asn in TM6 (N882) also helps bind the phosphate group and an early mutagenesis screen identified the crucial nature of this residue for substrate recognition in ATP8A2 (N905) [113]. PC occupies the same entry gate position in structures of Dnf1-Lem3 (Fig. 5). However, it remains unclear what entry gate features distinguish a GlcCer/PC/PE flippase, such as Dnf1 and Dnf2, from a flippase that only transports GlcCer (human ATP10D or Dnf2 from *Schizosaccharomyces pombe*) or strongly prefers PC over GlcCer (ATP10A) [55,70].

### 4.2. Substrate translocation - the hydrophobic gate

Directly above the entry gate binding site on TM2, 4, and 6 sits a ring of nonpolar residues surrounding the Pro +1 Ile in TM4 that forms the hydrophobic gate in the P4-ATPase structures. This hydrophobic gate helps occlude the lipid in the entry gate binding site and generates a barrier for further movement of the substrate along the TM2-4-6 groove. A mutation of the Pro +1 Ile residue in ATP8A2 (I364M) causes Cerebellar Ataxia, Mental Retardation, and Disequilibrium syndrome 4 (CAMRQ4), a striking neurological disorder where affected individuals have difficulty walking upright on two legs and often use a quadrupedal gait for mobility [116]. The ATP8A2 I364M mutation substantially diminishes the ability of PS to stimulate its ATPase activity and nearly ablates PS flippase activity [109]. Mutations of surrounding hydrophobic residues also perturb ATP8A2 function although not as substantially as changes to I364 [109]. Most of the amino acid side chains lining the cytosolic half of the TM2-4-6 groove are also hydrophobic. This leads to the key unanswered question of how the substrate lipid headgroup traverses this barrier to reach the cytosolic leaflet.

### 4.3. Substrate translocation - exit gate binding site

Surprisingly, the structures of Dnf1-Lem3 and Dnf2-Lem3 in the E2~P•PL $\left({\text{BeF}}_{3}^{-}\right)$ state from both *Saccharomyces cerevisiae* and *Chaetomium thermophilum* revealed lipid bound in a cytosolically exposed exit gate [96,97,117] (Fig. 5). This exit gate binding site uses residues from diverging segments of TM2 and TM4 helices that extend out of the membrane, the loop connecting TM4 to the P-domain, the first helix of the P-domain, and the N-terminus of the β subunit (Lem3 R51)(Fig. 5). The lipid in the exit gate binding site is about 10 Å out of the plane of the bilayer and likely dimples the cytosolic leaflet adjacent to this lipid [97]. Remarkably, mutations in the most membrane-distal residue in this binding site, Lem3 R51, alter the substrate specificity of Dnf2-Lem3 [96]. Evidence that this exit gate binding site is a conserved feature of P4-ATPases comes from studies of Neo1, ostensibly the most ancient extant member of the P4-ATPase family. Despite the absence of a β subunit, mutations in Neo1 residues lining the exit gate binding site disrupt transport and/or alter substrate preference [95]. For example, mutation of the membrane-distal R247 from the Neo1 TM2 helix, structurally equivalent to Dnf1 R264 shown in Fig. 5, specifically disrupts PE transport, as measured by a loss of PE asymmetry while the mutant cells maintain PS asymmetry. This type of separation-of-function mutation, which disrupts the transport of one substrate without noticeably perturbing the transport of a second substrate, can suggest direct substrate interaction. Similarly, mutations in the ATP8A2 exit gate (TM2; K94M and R102A) perturb PS transport [118]. Thus, it seems likely that the lipid present in the Dnf1/2-Lem3 exit gate is a substrate lipid and all P4-ATPases will dock substrate in this exit gate before releasing it into the cytosolic leaflet.

However, it is unclear why both entry and exit sites are occupied by lipids in the Dnf1/2-Lem3 cryoEM E2~P•PL structures (Figs. 4 and 5) when only a single lipid molecule is thought to be transported per ATP invested. An alternating half-channel transport model would imply that the exoplasmic entry gate opens while the exit gate is closed, substrate lipid would enter and be occluded as the entry gate closes behind it, and then the exit gate opens to allow release of substrate into the cytosolic leaflet. In contrast, the Dnf1/2-Lem3 entry and exit gates appear to be open to both the cytosolic and extracellular leaflets simultaneously in the E2P•PL $\left({\text{BeF}}_{3}^{-}\right)$ state. However, the hydrophobic gate between the lipids is closed and this should prevent the scrambling of lipids if this intermediate is present in native membranes. There is currently no ${\text{AlF}}_{4}^{-}$ stabilized structure of PC/GlcCer flippases with bound lipid substrate, so the influence of substrate occlusion in the entry gate on lipid association with the exit gate is unknown. What is the relationship of the two lipids in the E2P•PL state to the transport cycle? A speculative model is that a previously transported substrate lipid may move into the exit gate from the cytosolic leaflet (dotted arrow with a question mark in Fig. 4, step 6) while the entry gate site loads with a new substrate lipid. The “older” substrate lipid would subsequently be replaced by the new lipid that is being flipped as the entry gate closes and substrate moves past the hydrophobic gate. It is also possible that the ordered lipids at the exit gate are annular lipids and not substrate, and that exit gate mutations influence substrate selectivity by altering the conformation of the entry gate binding site. By this model, the exit gate binding site could be an allosteric modulator of substrate binding at the entry gate.

### 4.4. Substrate translocation pathway between entry and exit gate binding sites

**Fig. 6.**
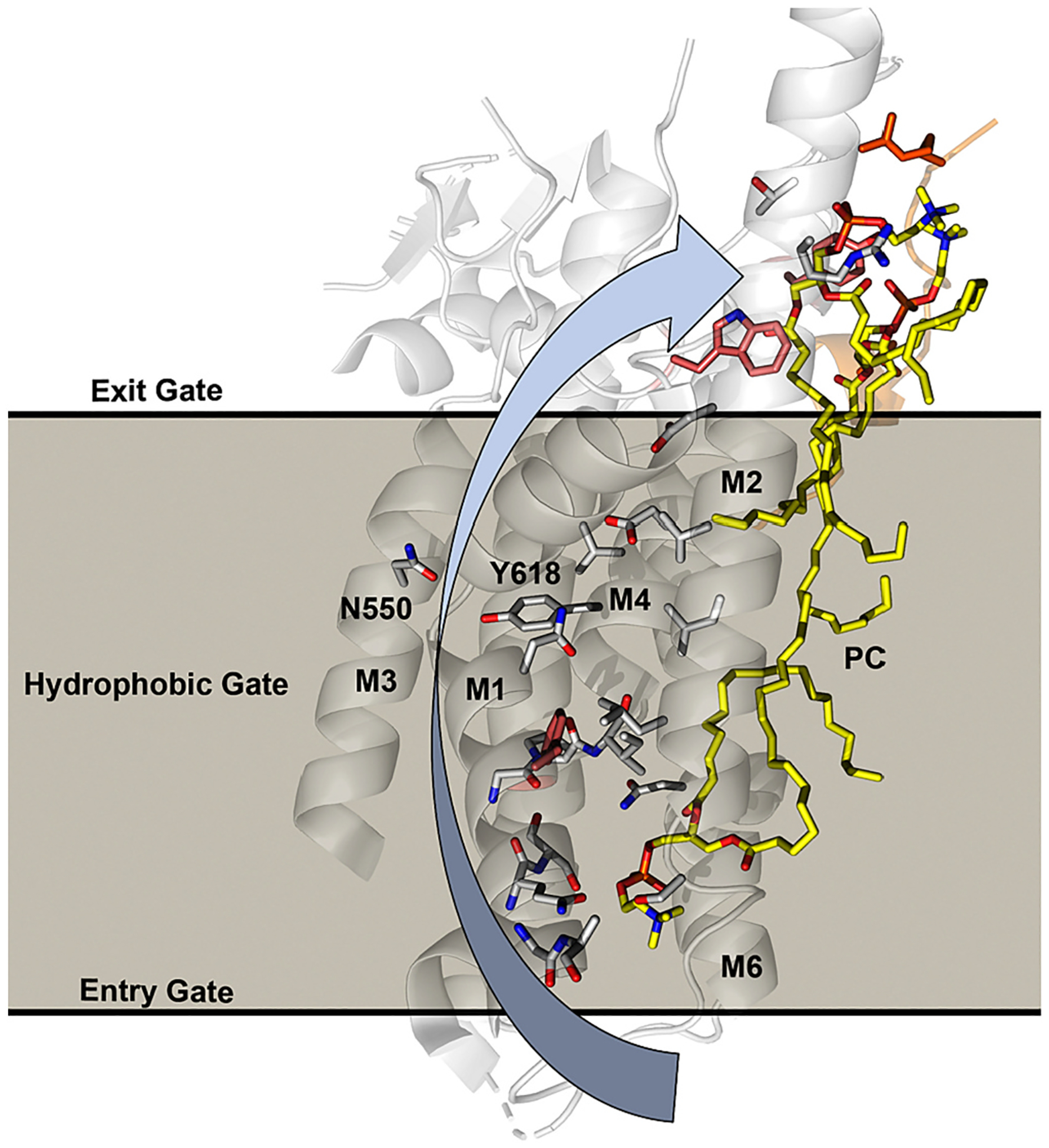
Potential substrate translocation path in the Dnf1-Lem3 membrane domain. The side chains are shown for residues where mutations alter the substrate preference of Dnf1-Lem3. N550 and Y618 are highlighted because these side chains do not line the TM2,4,6 groove, along which the headgroup is thought to slide during transport. However, N550 and Y618 could define part of the translocation pathway if TM1,2 partially separates from TM3,4,6 to allow the substrate headgroup to slide between them.

How do substrate lipids flip as they move from the entry gate binding site to the exit gate binding site? The “credit card” model suggests that the headgroup slides along the TM2-4-6 groove while the fatty acyl chains re-orient within the hydrophobic interior of the bilayer – comparably to swiping a credit card through a reader. However, the hydrophobic gate imposes a barrier to the sliding of the polar headgroup and none of the available structures have caught the substrate in an intermediate position between the two binding sites. Mutagenesis studies have implicated several residues that line a potential translocation pathway and argue against the simplest version of the credit card model. These include polar residues in TM1, TM3, and TM4 that do not line the TM2–4–6 groove and for which mutations substantially impact the substrate specificity (such as Dnf1 N550 and Y618 (Fig. 6)) [79,111,112]. The substrate headgroup could access the TM1–3–4 residues if TM1–2 briefly separates from TM3–10 to allow penetration of the headgroup within the membrane domain. In fact, substantial differences are observed in the positions of TM1 and TM2 when Dnf1-Lem3 $\left({\text{BeF}}_{3}^{-}\right)$ is reconstituted in yeast phospholipid bilayers prior to cryoEM relative to detergent-solubilized Dnf1-Lem3 $\left({\text{BeF}}_{3}^{-}\right)$ [117]. Thus, TM1–2 may be sufficiently mobile to allow substrate headgroups to move between TM1–2 and TM3–10. By this speculative model, the hydrophobic gate would interact with the substrate acyl chains as the lipid flips, while the headgroup would engage polar residues in the cleft formed from TM1–3–4. Alternatively, TM1–3–4 “out-of-groove” mutations may alter the conformation of the entry or exit gate binding pockets to modulate substrate specificity.

The mechanism for how substrate induces dephosphorylation is unclear. Substrate binding to the entry gate is a prerequisite and may be sufficient to induce dephosphorylation. For example, mutation of the TM6 Asn in the entry gate of ATP8A2 permits phosphorylation but blocks substrate-stimulated dephosphorylation [113]. However, lipids are found in both entry and exit binding sites when Dnf1/2-Lem3 is in the E2 ~ P conformation, and both these sites are formed in the E2 ~ P state and disrupted in the E1 state. Thus, it seems possible that dephosphorylation (E2 ~ P•PL → E2•PL) occurs during transport or upon arrival of the substrate at the exit gate. In this regard, it is intriguing that lipid in the exit gate docks against helix 1 of the P domain, which is closely linked to the aspartyl~phosphate group (Figs. 3 and 5). In Neo1, mutation of the first residue in helix 1 (S488W or S488A) of the P-domain strongly disrupts both PE and PS membrane asymmetry [96]. Thus, it will be important to determine how S488 mutations influence the kinetics of Neo1 phosphorylation and dephosphorylation. Interestingly, an Ile to Ala mutation at the ATP8A2 Pro +1 position in the hydrophobic gate appears to stabilize the dephosphorylated E2•PS state and slows transport prior to the electrogenic (current producing) transport step for this negatively charged substrate [118]. The electrogenic step is linked to dephosphorylation but it remains to be determined if it is the flipping event, release of PS to the cytosolic leaflet, or a combination of both steps that produces the current. Interestingly, one of the TM2 exit gate mutations tested (K94M) strongly abrogates the electrogenic step while a second (R102A) significantly slows the kinetics of this step [118]. Further studies are needed to fully understand how the energy landscape of substrate translocation is linked to conformational changes and flippase dephosphorylation.

### 4.5. P4-ATPase regulation

The Drs2 C-terminal tail is an autoinhibitory domain and regulatory factors bind this tail to relieve autoinhibition and stimulate Drs2 activity [104,105,119]. CryoEM structures of Drs2 reveal that autoinhibition is provided by the interaction of C-terminal tail residues 1252–1307 with the N- and P-domains [94,119]. Residues 1250–1270 compose an Arf-GEF (Gea2) binding site and interact with the P-domain while a conserved GFAFSQ (1274–1279) motif binds the N-domain thus inhibiting Drs2p. A basic patch between the ArfGEF binding site and GFAFSQ motif is important for Drs2 function and binds to phosphatidylinositide 4-phosphate (PI4P), a crucial activator of Drs2 [105]. PI4P also binds along TM10 with the headgroup interacting with a membrane-proximal helix in the C-terminal tail [94]. PI4P partially relieves autoinhibition of Drs2p by disrupting the interaction between the C-terminal tail and the P-domain. Full displacement of the C-terminal tail will allow Drs2p to become fully active and this depends on auxiliary proteins such as the Arf-like protein Arl1 and the ArfGEF Gea2. Arl1 binds to the Drs2 N-terminal tail and to Gea2 and this ternary complex is required for full activation of Drs2 [120]. The F-box protein Rcy1 binds to the same Drs2 C-terminal sequences as Gea2 and also appears to activate Drs2 [121]. These regulatory interactions likely exist to restrict Drs2 activity to the correct location in the cell (the TGN or early endosome) and temporally when there is a high demand for vesicular transport from the Golgi, such as periods of rapid growth.

Similar to the Drs2 GFAFS motif, Atp8B1 contains a C-terminal AYAFS motif which when expressed as a peptide can inhibit Atp8B1 ATPase activity [84]. However, this inhibitory activity was prevented by phosphorylation of a conserved S1223 residue, which was originally identified in large-scale phosphorylation proteomic studies. In addition to the C-terminal inhibition, the removal of the N-terminus of Atp8B1 further increases Atp8B1 activity which suggests that the N- and C-terminal tails cooperatively inhibit Atp8B1. Furthermore, it was found that phosphoinositides, particularly PI(3,4,5)P_3_, potently stimulate ATPase activity. PI(3,4,5)P_3_ is synthesized by phosphoinositide 3-kinase (PI3K), which can be activated by several different bile acids [122]. In addition, ATP8B1 ATPase activity is directly stimulated by bile acids [86].

The yeast Dnf1 and Dnf2 are directly phosphorylated and activated by Flippase Protein Kinases (Fpk1 and Fpk2), which are part of a TORC2 membrane homeostatic network [123,124]. TORC2 acts upstream of Fpk1 by activating Ypk1 which in turn phosphorylates and inactivates Fpk1 [124,125]. In addition to Ypk1, Gin4 is a kinase that adds inhibitory phosphates to Fpk1 independent of TORC2 activation [126]. A phosphoprotein phosphatase 2A homolog (Sit4-Sap190) is needed to remove the inhibitory phosphates on Fpk1, thereby increasing Dnf1 and Dnf2 phosphorylation and flippase activity [126]. In addition to plasma membrane asymmetry, the TORC2-Ypk1 axis also regulates sphingolipid synthesis and endocytosis to help balance membrane composition and organization [125,127]. The structural basis for how FPK-dependent phosphorylation stimulates Dnf1/Dnf2-Lem3 flippase activity is unknown. However, Dnf1 can adopt an inactive, “resting” conformation with the A domain lying flat against the membrane when it is reconstituted in a yeast lipid bilayer [117]. Interestingly, three of the six FPK phosphorylation sites lie within an unstructured loop in the A domain [128], suggesting that phosphorylation may help disrupt interaction of the A domain with the membrane to promote activity.

Caspases are a family of proteases which play a central role in apoptosis, and one feature of apoptosis is increased PS exposure on the plasma membrane. To accomplish this dynamic change in membrane asymmetry, plasma membrane scramblases must be activated while flippases must be inhibited. Indeed, the TMEM16F scramblase is activated by a calcium influx and the Xkr8 scramblase is activated by caspases during apoptosis [27,28,48]. Conversely, ATP11A and ATP11C are inhibited by high concentrations of cytosolic calcium or caspase cleavage [89]; Atp11A and Atp11C contain two caspase protease target sites between the P- and N-domains. Mutations of the caspase protease sites in ATP11C resulted in reduced PS exposure during FasL-mediated induction of apoptosis [89,129]. These results indicate that PS flippases can be regulated by protease cleavage from the caspase protein family, which is an important step for apoptotic cell recognition by professional phagocytes.

## 5. P4-ATPase function in protein trafficking

The first link between a P4-ATPase and protein trafficking came from a genetic screen in *Saccharomyces cerevisiae* designed to identify mutations that disrupt protein transport from the Golgi. This screen uncovered a requirement for Drs2 to bud clathrin-coated vesicles from the *trans*-Golgi network (TGN) [35]. As additional yeast P4-ATPases were identified and functionally characterized, it became clear that they all contribute to various protein trafficking events in the secretory and endocytic pathways [36,37,41,54,69,98,130–133]. These include a requirement for Neo1 in COPI-dependent protein transport from the Golgi and retromer-dependent transport from endosomes [37,42,134,135], Drs2 in budding AP-1/clathrin-coated vesicles from the TGN and recycling vesicles from endosomes [36,56,131,136], redundant functions for Drs2 and Dnf1/2 in budding GGA/clathrin and AP-3-coated vesicles from the Golgi [36], and Dnf1/2 in endocytosis and endosomal recycling [7,36,69]. Orthologs of Drs2 in *C. elegans* (TAT-1) and mammals (ATP8A1, ATP8A2) are also required for Golgi-endosomal trafficking [43,64,137,138], and Neo1 orthologs (TAT-5/ATP9A) are linked to endosomal recycling [58,139–141]. In addition to disrupting intracellular vesicle trafficking, TAT-5 and ATP9A deficiency cause a substantial increase in extracellular vesicle release from the plasma membrane of cells [58,59,142]. How TAT-5 and ATP9A normally suppress extracellular vesicle release is currently unclear.

The flippase requirement for particular trafficking events is primarily determined by the specific transport substrate and the influence of lipid transport on membrane curvature. For example, Drs2 is required for bidirectional protein transport between the TGN and early endosome. None of the other yeast P4-ATPases can normally compensate for the loss of Drs2 in these pathways, even though they all traffic through the Golgi and endosomes. However, mutations in the Dnf1 or Neo1 substrate translocation pathway that enhance their ability to flip PS allow these flippases to significantly replace Drs2 trafficking functions in the cell [56,114,143]. The curvature and charge imparted to the cytosolic leaflet by a PS flippase activity can be recognized by specific effector proteins. In yeast, for example, the ArfGAP Gcs1 contains an ArfGAP Lipid Packing Sensor (ALPS) motif that recruits this protein to curved, PS-rich membranes established by Drs2 or the Dnf1[PS] variants [56]. In mammals, EHD1 and evectin-2 are protein trafficking effectors of PS enriched in the cytosolic leaflet of endosomes by ATP8A1 [43,44,144,145]. Unidirectional lipid transport creates an imbalance in surface area between the two leaflets that promote membrane bending in the direction of lipid transport (towards the cytosol) [146], and both gain-of-function and loss-of-function phenotypes support this hypothesis for how P4-ATPases support vesicle budding [35,56,87,147].

## 6. P4-ATPase disease associations

**Fig. 7.**
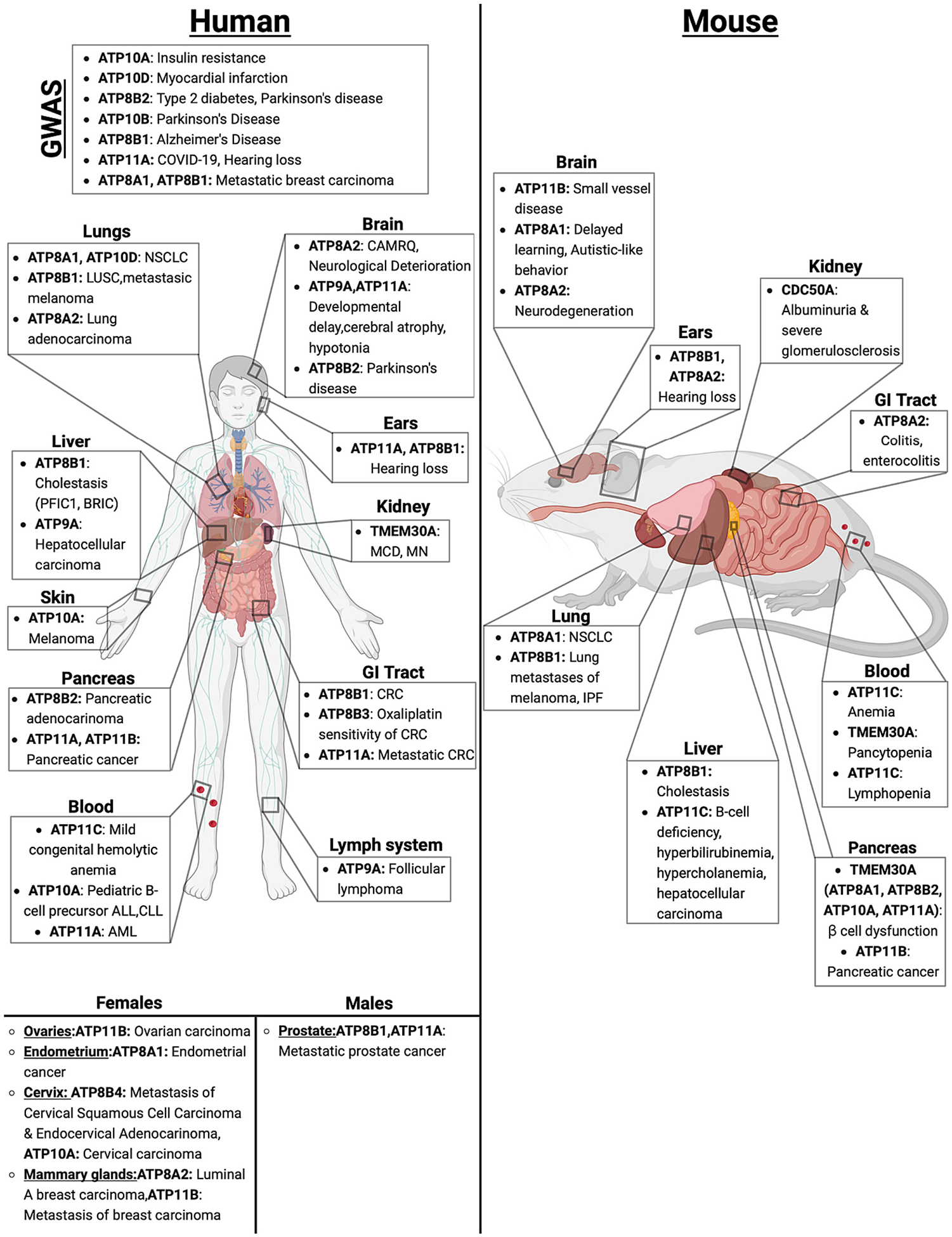
Involvement of P4-ATPases in human and mouse pathologies. The contribution of P4-ATPases to various pathologies in humans and mice can affect several locations in the body; including the lungs, brain, liver, skin, kidney, pancreas, GI tract, ears, blood and lymph, as well as female and male reproductive organs. Refer to the text for further description and citations. Schematic created using Biorender.com. NSCLC = Non-small cell lung cancer, LUSC = lung squamous cell carcinoma, PFIC1 = progressive familial intrahepatic cholestasis type 1, BRIC1 = benign recurrent intrahepatic cholestasis type 1, MCD = minimal change disease, MN = Membranous nephropathy, CRC = Colorectal cancer, ALL = Acute lymphocytic leukemia, CLL = Chronic lymphocytic leukemia, AML = acute myeloid leukemia, IPF = Idiopathic pulmonary fibrosis, CAMRQ = Cerebellar ataxia, impaired intellectual development, and disequilibrium syndrome.

P4-ATPases have been found to play a role in a wide range of diseases in both humans and mice, including metabolic disorders such as cholestasis and type 2 diabetes, anemia, neurological disorders, and several different kinds of cancer (Fig. 7).

### 6.1. Liver

The action of ATP8B1, which appears to be a PS/PC transporter [81,86,87], is important in bile acid homeostasis in humans. Mutations in the *ATP8B1* gene lead to impaired bile flow, also known as cholestasis, which can result in extensive liver damage. Two forms of inherited cholestasis, benign recurrent intrahepatic cholestasis type 1 (BRIC1) and progressive familial intrahepatic cholestasis type 1 (PFIC1) were previously mapped to 18q21 and haplotype analysis was then used to identify the mutated gene as *FIC1* (familial intrahepatic cholestasis 1), also known as *ATP8B1* [148]. Moreover, *Atp8b1*-deficient mice display an increase in the biliary extraction of cholesterol from the canalicular (apical) membrane of hepatocytes, affecting the activity of the bile salt transporter, ABCB11, and resulting in cholestasis [149]. The etiology of ATP8B1-associated cholestasis has been extensively covered elsewhere [150,151].

The structure of ATP8B1 was recently determined, allowing for the structural implications of disease-causing mutations in this flippase to be further analyzed [85,86]. The PFIC1 and BRIC1 mutations are homogeneously distributed along the ATP8B1 amino acid sequence, however, two mutations, D445N and H535L, in the nucleotide-binding pocket could potentially alter ATP binding. Additionally, the D445 residue is at an interacting distance with the autoinhibitory C-terminus so its alteration could potentially affect the regulation of ATP8B1. These authors also found several mutations in the P-domain that would abolish autophosphorylation of the catalytic aspartate (D454) and result in an inactive flippase; these mutations include S453Y, D454G, and T546M. Some PFIC1 patients exhibit an S403Y mutation in the TM4 PISL motif that is conserved in most P4-ATPases. Mutation at this residue could potentially diminish ATPase activity and substrate affinity.

There is also evidence for ATP11C, a PS flippase, having a role in bile acid homeostasis. *Atp11C* deficiency in mice causes hyperbilirubinemia and cholestasis [152]. These pathologies could arise in *Atp11C* deficient mice because ATP11C is essential for basolateral membrane localization of multiple bile salt transport proteins in hepatocytes, which mediate the transport of bile acids and organic anions across the sinusoidal membrane [153,154]. Additionally, GWA studies have implicated *ATP10A* and *ATP10D* in insulin resistance, HDL homeostasis, and atherosclerosis, suggesting that these flippases influence liver metabolism [155–157]. The commonly used C57BL/6 J inbred mice have a premature stop codon in the middle of the *Atp10D* open reading frame and are naturally deficient for this protein [158]. Transgenic C57BL/6 J bearing a single copy of the wild-type Atp10D displayed improved metabolic parameters after being fed a high-fat diet, such as lower plasma triglycerides, lower fasting glucose, and improved insulin sensitivity [159]. The mechanism for how GlcCer transport by ATP10D influences metabolism and atherosclerosis remains unclear.

### 6.2. Pancreas

One method to determine how P4-ATPases generally affect a biological process is to create CDC50A/TMEM30A knockouts and measure the resulting phenotypes. This methodology does not elucidate which specific flippase(s) are important in a process, however, it does show how globally perturbing P4-ATPase localization and function can affect different biological processes across many tissues and cell types. This approach was used to discover that P4-ATPases play a role in insulin secretion and glucose sensing in pancreatic β cells [160]. The authors created a mouse line where *Tmem30a* was knocked out specifically in pancreatic β cells and found that the mice exhibited glucose intolerance that worsened with age, abnormally large islets with increased beta cell mass, and hyperglycemia with impaired glucose-stimulated insulin secretion. Additionally, using islet cell culture models, they found that *Tmem30a* deficiency led to impairments in insulin maturation, the clathrin-dependent formation of mature insulin secretory granules, and less GLUT2 at the surface of β cells.

### 6.3. Red blood cells

The history of how erythrocytes were utilized to discover lipid flippases nearly 40 years ago has been extensively described elsewhere [7]; however, the in vivo implications of this enzyme’s activity in red blood cells are still being explored. ATP8A1 was originally thought to be responsible for the majority of the flippase-mediated PS translocation in murine erythrocytes [161], however, it was later found that *Atp8a1*^−/−^ mice do not display increased PS exposure on their erythrocytes, most likely due to compensation by other flippases [162]. One such compensatory flippase is ATP11C, one of the most abundant flippases in both human and mouse red blood cells [77,163]. *Atp11c* deficiency causes anemia in mice [164] and multiple erythrocyte abnormalities; including elevated PS exposure, shortened life span, and abnormal shape [165]. These abnormalities may be due to implications of the bilayer couple hypothesis [18,166,167] and/or altered interaction with PS and skeletal proteins at the plasma membrane [168,169]. Additionally, conditional KO of *Tmem30a* in hematopoietic cells results in pancytopenia in mice [170], further highlighting the importance of proper function and localization of flippases to the maintenance of blood cell homeostasis.

Phenotypes that arise due to mutations in ATP11C support the importance of this flippase in maintenance of proper erythrocyte function. A Thr418Asn (T418N) mutation in ATP11C, inherited as an X-linked recessive trait, was found in a male patient with mild congenital hemolytic anemia and resulted in a 10-fold decrease of PS internalization in erythrocytes compared to controls [163]. The equivalent Thr415Asn (T415N) mutation in mice, close to Asp409, the phosphorylation site for the formation of the phosphoryl intermediate, was shown to result in a 61 % decrease in ATPase activity and decreased ATP11C expression due to protein misfolding and ER retention [77,171]. Taken together, modulating plasma membrane PS localization via flippase activity can have broad effects on erythrocyte longevity and thus the body’s capacity to effectively deliver oxygen to tissues.

There is also evidence that ATP11C’s role in maintaining PS in the inner leaflet is more significant in senescent erythrocytes; there is lower expression of this flippase in senescent erythrocytes and decreases in intracellular [K^+^] and [ATP] as well as increases in [Ca^2+^] and plasma membrane tubulin [172]. These changes result in reduced ATP11C activity and consequently increased exposure of PS in senescent erythrocytes [172]. Additionally, normal erythrocytes incubated with the PKCζ activator, phorbol myristate acetate (PMA), displayed higher PS exposure compared to sickle-shaped erythrocytes incubated with the same drug [173]. PMA also induces tubulin polymerization, which negatively affects flippase activity [174]. Overall, negative regulation of flippase activity, likely ATP11C, contributes to PS exposure during erythrocyte senescence. ATP11C is also regulated in non-senescent cells; following treatment with PMA in human and mouse cell lines, ATP11C undergoes clathrin-mediated endocytosis via Ca^2+^-induced PKC activation [175]. This sequesters the protein and acts as a negative regulatory mechanism.

The importance of ATP11C in erythrocyte homeostasis in mice versus humans is still being explored. In mice, ATP11C mutations result in several phenotypes, including B-cell deficiency, anemia, hyperbilirubinemia, cholestasis, and hepatocellular carcinoma [152,153,164,176,177]. However, the phenotypes seen in humans due to *ATP11C* mutation are much milder, perhaps because there is some residual activity of the mutant ATP11C in erythrocytes, there is compensation by other lowly expressed erythrocyte flippases (ATP11A and ATP11B) [77], or maybe, ATP11C has a more important role in mouse physiology versus humans and this is still an open area of research. It was recently discovered that erythrocytes treated with a microtubule-stabilizing chemotherapy drug, Paclitaxel, exhibit PS exposure, most likely due to the drug increasing the amount of tubulin at the plasma membrane, thus inhibiting flippase activity [174]. There is also an increase in PS exposure in erythrocytes from people with diabetes and hypertension, conditions that also result in changes to tubulin dynamics and post-translational modification [178–180]. Interestingly, Paclitaxel treatment can lead to various vascular complications such as stroke, deep vein thrombosis, and pulmonary embolism. Moreover, anemia and hypercoagulability are frequently observed in diabetic and hypertensive patients, respectively [181,182]. Further exploration is needed to determine the role of flippase dysregulation and deficiency to the underlying causes of hemorheological dysfunctions in cancer patients receiving microtubule-targeting drugs (such as Paclitaxel), and in diabetic and hypertensive patients. This investigation could shed light on the potential role erythrocyte flippases have in vascular health in humans.

### 6.4. Immune system

ATP8A2 plays an important role in the survival of Intestinal intraepithelial lymphocytes (TCRαβ+CD8αα + T cells) and these cells are needed to suppress colitis [183]. Mice deficient for Notch signaling in T-cells displayed a substantial reduction in intestinal intraepithelial lymphocytes and the surviving cells expressed very low levels of ATP8A2. This resulted in PS exposure in the outer leaflet of the plasma membrane of nonapoptotic cells, which were engulfed by intestinal macrophages. Thus, membrane phospholipid asymmetry controlled by Notch-mediated ATP8A2 expression is critical for maintaining the appropriate number of Intestinal intraepithelial lymphocytes.

Flippases were shown to be essential in the inflammatory response by mediating the endotoxin-induced endocytic retrieval of Toll-like receptor 4 (TLR4) in human primary monocyte-derived macrophages [184]. In these cells, depletion of CDC50A resulted in hypersecretion of proinflammatory cytokines, increased MAP kinase signaling and constitutive NF-κB activation in response to endotoxin. These phenotypes were recapitulated by separate knockdown of ATP8B1 and ATP11A suggesting an important role for these flippases in the innate immune response.

Mutations in *Atp11c* cause X-linked B cell-deficiency syndrome in mice. ATP11C catalyzed PS flippase activity in developing B-lymphocytes, and was essential for normal B cell differentiation from the pro-B cell stage onwards, while not affecting T cells [164]. ATP11C-deficient pre-B cell proliferate normally in response to IL-7 but failed to differentiate into immature B cells upon removal of IL-7, suggesting that regulation of PS asymmetry may control the switch from proliferation to differentiation in pre-B cells [185]. ATP11C in precursor B cells is essential for rapidly internalizing PS from the cell surface to prevent the cells’ engulfment by macrophages and avoid severe B-cell deficiency (B-cell lymphopenia) [177].

### 6.5. Kidney

A very limited amount of experimental work has been reported on P4-ATPases in kidney disease. Of the 12 P4-ATPases that associate with CDC50A subunits, 5 P4-ATPases (ATP8A1, ATP11A, ATP11B, ATP11C and ATP10D) were detected in mouse kidney [103]. The phosphatidylserine flippase β-subunit TMEM30A (CDC50A) is highly expressed in human glomeruli. Significantly reduced TMEM30A expression was detected in the glomeruli of patients with MCD (minimal change disease) or MN (membranous nephropathy) [186]. In mice, *Tmem30a* is essential for the survival and function of podocytes, and podocyte-specific deletion of *Tmem30a* results in albuminuria and severe glomerulosclerosis [186]. Since MCD and MN are characterized by proteinuria and podocytopathy, this implies that TMEM30A is essential for kidney health.

### 6.6. Metabolism and metabolic signaling

There is evidence for several flippases having roles in metabolism in humans. SNPs in *ATP10A* have been shown to correlate with an increased risk of insulin resistance in a cohort of African Americans [155]. Additionally, *Atp10A* deficiency in mice may result in glucose intolerance, reduced insulin sensitivity, and hyperinsulinemia [187–189]. However, the mouse model used for these studies carried a large chromosomal deletion and whether these phenotypes arise from *Atp10A* deficiency or the loss of multiple genes in the deletion interval remains to be determined. SNPs in *ATP10D* are associated with increased circulating levels of hexosylceramides (GlcCer and/or galactosylceramide) and increased incidences of myocardial infarction in European populations [157]. One of these *ATP10D SNPs* was found to associate with increased atherosclerotic severity and to modulate HDL abundance in an elderly Japanese population [156]. This increase in plasma hexosylceramide levels in people with *ATP10D* SNPs was quite mysterious until our group discovered that ATP10D translocates GlcCer [70]. C57BL/6 J mice, a popular mouse strain used in metabolic studies due to their susceptibility to metabolic disorders [190], have a premature stop codon in *Atp10D* and is therefore an *Atp10D* null mouse [158]. Reintroducing *Atp10D* into C57BL/6 J mice results in mice having a more favorable metabolic profile on a high-fat diet, characterized by less weight gain, lower plasma triglyceride, glucose, insulin and hexosylceramide levels, and improved whole-body insulin sensitivity [159]. Additionally, *ATP8B2* has also been identified as a susceptibility locus for type 2 diabetes [191]. ATP8B2-mediated establishment of an asymmetric distribution of plasmalogens in the plasma membrane affects the phosphorylation and activation of AKT, and thus cell metabolism and growth [90].

### 6.7. Nervous system

The nervous system is uniquely sensitive to mutations in P4-ATPases, as a majority of the P4-ATPases are expressed in the brain, spinal cord, and sensory organs including the retina and hair cells of the inner ear. Mutations in ATP8A2 are well described as they cause CAMRQ4 as described in Section 4.2 [116,192,193]. A total of 33 patients have been documented with mutations in ATP8A2 and with that, the clinical definition has been broadened to include developmental delay, movement disorders, and optic atrophy [194,195]. Patients with strongly inactivating mutations, such as a premature stop codon or N917D in the entry gate, cannot walk, crawl, sit, or feed themselves [196]. Ataxia, axonal degeneration and hearing loss are also observed in the wabbler-lethal mouse and these phenotypes are caused by an *Atp8A2* mutation [197,198]. Similarly, mutations in ATP9A and ATP11A cause severe developmental delay, cerebral atrophy, and hypotonia [115,139,199,200]. The cause of cerebral atrophy has been linked to the loss of dendrites and subsequent synapses in pyramidal cells in both the cortex and hippocampus. The mechanism of neuronal loss is likely due to exposure of PS along the axon and dendrites, leading to the engulfment of these appendages by microglia. ATP8A2 and ATP11A flip PS and while the ATP9A substrate has not been verified, the homolog Neo1 flips PS as well, suggesting that PS flippases are key for avoiding neuronal loss. This is corroborated by studies showing that flippase downregulation is necessary for dendrite pruning in the development of the nervous system [201,202]. In addition to controlling PS asymmetry, ATP11B in mice has shown to regulate synaptic plasticity both by increasing glutamate release and modulating the expression of glutamate receptors [203]. Additionally, *Atp8a1* deficiency in mice results in an increase in PS externalization in hippocampal cells; leading to dysfunctional hippocampus-dependent learning [162]. ATP8A1 was also found to be induced in post-mortem tissue homogenates from the hippocampus and temporal lobe of autistic subjects and a mouse model with elevated levels of ATP8A1 displayed weaker excitatory synapses in the hippocampal CA1 region and aberrant social behavior; indicating that both elevated and diminished levels of ATP8A1 during early development are detrimental to brain connectivity [204].

P4-ATPases could play a significant role in preventing the progressive neurodegenerative diseases Parkinson’s and Alzheimer’s. Mutations in *ATP10B*, a GlcCer flippase that localizes to the *endo*-lysosomal system, were found in a cohort of Parkinson’s disease (PD) patients [82]. These authors hypothesized that mutations in ATP10B might lead to altered lysosomal export of GlcCer and this could be detrimental to neuronal health. However, it remains controversial whether ATP10B is associated with PD due to limitations in sample size and low prevalence of the variants [205–207]. *ATP8B1* was associated with resilience to Alzheimer’s Disease (AD) based on the observation that several SNPs within the *ATP8B1* enhancer that are enriched in asymptomatic individuals compared to AD patients. This is an interesting connection because ATP8B1 manages bile acid homeostasis, and increased bile acid levels have recently emerged as a potential biological contributor and biomarker for AD [208]. Variants within *ATP8B2*, a plasmalogen transporter, are associated with motor deficit progression of PD-afflicted patients [209], and reduced levels of plasmalogens cause a human genetic disorder, rhizomelic chondrodysplasia punctata (RCDP), characterized by severe neurological symptoms [210]. The role of ATP8B2 in the translocation of plasmalogens should be further examined in the context of both RCDP and PD progression.

P4-ATPases can be expressed in nervous system cell types other than neurons; including astrocytes, microglia, oligodendrocytes as well as the vasculature including pericytes and vascular endothelial cells. ATP11B is expressed in the vascular endothelium of the brain and is needed for the development of the endothelium cell layer by inducing the formation of tight junctions [211,212]. In addition, loss of ATP11B causes endothelium cell dysfunction via decrease in eNOS signaling and within retinal vessels has been shown to reduce the diameter and increase branching of the vasculature. From this, ATP11B could contribute to brain vasculature diseases such as cerebral small vessel disease and cerebral amyloid angiopathy [213].

### 6.8. Lung

Patients with *ATP8B1* mutations causing progressive familial intrahepatic cholestasis (PFIC) appear to be susceptible to pneumonia [214], and mice homozygous for the *Atp8a1-G308V* PFIC mutation were more sensitive to bacteria-induced lung injury [84]. A study of global gene profiling of aging lungs in *Atp8b1* mutant mice suggested links to oxidative damage and fibrosis [215]. Exposure of these mice to oxidative stress (hyperoxic conditions) led to idiopathic pulmonary fibrosis due to accelerated lung epithelial cell apoptosis in alveoli and augmented proliferation of oxidative stress-resistant club cells [216,217]. ATP8A1 has also been linked to lung function because it is a critical cargo of AP-3 vesicles involved in lamellar body biogenesis within alveolar type 2 cells. AP-3 mutations, as occur in Hermansky Pudlak syndrome, cause accumulation of ATP8B1 in endosomes, where excessive PS transport hyperactivates Yes-activated protein (YAP) signaling to promote fibrosis [218]. Another study found an association between variants in *ATP11A* and poor outcomes for COVID-19 patients. Immune-mediated inflammatory lung injury is a main cause of critical COVID-19. The GenOMICC (Genetics of Mortality in Critical Care) study compared genomes from individuals who were critically ill to those who were asymptomatic or with mild symptoms. Using this resource (7491 critically ill and 48,400 controls), *ATP11A* was discovered as one of 23 independent gene variants leading to reduced expression that significantly predispose to critical COVID-19 [219].

### 6.9. Cancer

A role for ATP8A1 in the development and progression of non-small-cell lung cancer (NSCLC) was discovered in a study of the tumor suppressor function of microRNA MiR-140–3p. The growth of NSCLC cells in nude mouse models was suppressed by overexpression of miR-140–3p which was attenuated by overexpression of ATP8A1 [220]. In a study of 25 cases of tumor tissues and the adjacent normal tissues from surgeries of NSCLC patients, ATP8A1 was found to be overexpressed in NSCLC tissues by immunohistochemical staining [221]. Mechanistically, ATP8A1 promoted the expression of MMP-9 and Vimentin as well as suppressed the expression of E-cadherin thus resulting in the elevated invasion/migration ability of NSCLC cells [221].

In addition, *ATP8A1* was identified as a candidate gene involved in endometrial cancer predisposition, which could help in personalized prognosis [222].

*ATP8A1* and *ATP8B1* single nucleotide polymorphisms (SNPs) were associated with incurable metastatic breast cancer [223]. In addition to these P4-ATPases, findings from this study included other proteins that translocate and metabolize phospholipids entering the phosphatidylinositol cycle, which controls endo−/exocytosis. Another study of differentially expressed genes implicated *ATP8A2* as one of 5 key prognostic genes for individuals at high-risk for invasive breast cancer [224]. Additional differential gene expression analyses have implicated the reduced expression of *ATP8A2* in the progression of lung adenocarcinoma [225,226].

*ATP8B1* was initially identified as a driver gene for sporadic colorectal cancer (CRC) by the Genomic Identification of Significant Targets In Cancer (GISTIC) project [227] and several other studies support this association [228–230]. ATP8B1 was suggested to be a tumor suppressor for CRC, since the reduction of ATP8B1 expression either by CRISPR/Cas9 or shRNA was associated with increased growth and proliferation of CRC cell line HT29; while overexpression of ATP8B1 resulted in reduced growth and proliferation of SW480 cell lines [230]. In addition, the closely related *ATP8B3* was proposed to be a regulator of the oxaliplatin response in treatment of CRC and survival of the patients [231,232]. Most recently, ATP8B1 was identified as a prognostic biomarker for prostate cancer and lung squamous cell carcinoma, and that a higher ATP8B1 expression was associated with a better patient prognosis [233,234]. In LUSC cell lines, ATP8B1 knockdown promoted proliferation, inhibited apoptosis, and aggravated invasion and migration [234]. *ATP8B2* has been linked to the immune response to pancreatic cancer progression [235,236]. Interestingly, missense mutations in *Atp8b4* was associated with a striking increase in lung metastatic activity in a mouse melanoma model, which was consistent with transcriptome data from the human Cancer Genome Atlas predictive of improved patient survival [237]. *ATP8B4* was also one of 9 genes linked with metastasis in cervical carcinoma (20) [238]. Thus, the ATP8 clade of P4-ATPases shows a strong association with a variety of cancers, and more research is needed to understand how these flippases contribute to uncontrolled cell growth and metastasis.

High ATP9A expression levels correlate with poor outcomes for patients with hepatocellular carcinoma [239]. In hepatocellular carcinoma cells, ATP9A regulates micropinocytosis to promote nutrient acquisition and also interacts with a V-ATPase subunit (ATP6V1A) to facilitate its endosome to plasma membrane trafficking. These events, through an unknown mechanism, contribute to plasma membrane cholesterol accumulation and RAC1 activation to induce micropinocytosis and nutrient scavenging [239]. In contrast, under-expression of *ATP9A* was reported in relapsed follicular lymphoma patients [240], although no information as to how this influences cancer cell growth was reported.

ATP10A mutations have been linked to relapse of leukemia following chemotherapy. Recurrent aberrations of 14 genes were detected in patients with childhood acute lymphoblastic leukemia (ALL) who subsequently relapsed, including deletions or uniparental isodisomies of *ATP10A* [241]. A similar study to identify mutations enriched before and after therapy in patients with relapsed chronic lymphocytic leukemia (CLL) found that *ATP10A* mutations were enriched after relapse [242]. These results suggest that *ATP10A* mutations either promote the survival of cells exposed to chemotherapeutic agents or promote the growth of those cells that do survive. In addition, epigenetic increases in *ATP10A* methylation have been associated with the progression from normal through precancerous lesions to cervical cancer [243]. Lastly, coexpression network analysis and whole exome sequencing from melanoma-prone families identified *ATP10A* as a risk factor for cutaneous malignant melanoma [244].

*ATP10D* was specifically identified by a GWAS as a positive biomarker in individuals at high risk to develop tobacco-induced lung cancer [245]. Heavy smokers who developed non-small cell lung cancer (NSCLC) at an early age were compared to elderly smokers who were NSCLC-free to identify SNPs in *ATP10D* and *PDE10A* correlating with these extreme phenotypes. Moreover, a significant correlation of low mRNA expression of *ATP10D* with shorter survival in patients with stage I–II NSCLC supported the *ATP10D* prognostic value [245].

Differential methylation of *ATP11A* provides a prognostic biomarker for metastatic colorectal cancer and metastatic-lethal prostate cancer [246–248]. High methylation levels of CpGs in *ATP11A* also correlated with loss of the tumor suppressor gene *PTEN* which has an unfavorable prognosis in prostate cancer [249]. Further, low levels of *ATP11A* expression/methylation were identified as independent prognostic factors for acute myeloid leukemia (AML) [250,251]. In a comparison of pancreatic cancer and paracancerous tissues, ATP11A mRNA and protein levels were significantly higher in the cancer cells. Moreover, ATP11A promoted the invasion and migration of cultured pancreatic cancer cells via TGFβ-dependent epithelial-mesenchymal transition (EMT) [251].

High expression of *ATP11B* correlates with high-grade tumors in human ovarian cancer samples and with cisplatin resistance in ovarian cancer cell lines, for which sensitivity can be restored by silencing *ATP11B* [252]. Mechanistic studies in cell lines suggested that ATP11B localizes to the Golgi, where it contributes to the formation of secretory vesicles that deliver cisplatin from the Golgi to the plasma membrane for efflux [252]. In contrast, another study reported that levels of *ATP11B* were decreased in ovarian cancer tissues [253], suggesting that cisplatin therapy may select for the cells expressing higher levels of ATP11B from a population of low-expressing cancer cells. In addition, ATP11B was recently identified as an inhibitor of breast cancer metastasis. A CRISPR/Cas9 screen for gene mutations that promote metastasis was performed using mice carrying a mammary-specific knockout of *Brca1* [254]. This study demonstrated that low expression of ATP11B, a PS flippase, in conjunction with high expression of PS synthase induced by the *Brca1* deletion, caused exposure of PS on the outer leaflet of nonapoptotic breast cancer cells. The exposed PS is anti-inflammatory and immunosuppressive, which appears to provide a permissive tumor microenvironment that markedly accelerates tumor metastasis and associates with poor prognosis.

ATP11B was identified as a potential target of LTX-315, an oncolytic peptide deriving from bovine lactoferrin, and a critical regulator in maintaining Programmed cell Death Ligand 1 (PD-L1) cell surface expression in pancreatic cancer cells [255]. Elevated ATP11B levels correlate with poor outcomes in pancreatic cancer and several other cancers. Depletion of ATP11B in mouse pancreatic cancer cells strongly enhanced the survival of immunocompetent mice but had no effect on the survival of immunocompromised mice. The influence of ATP11B on cancer cell immune surveillance was traced to its control over the trafficking of PD-L1, which was mislocalized to the lysosome and degraded in ATP11B deficient cells. ATP11B interacts with PD-L1 through an adaptor protein (CMTM6), and these interactions are critical for recycling PD-L1 back to the plasma membrane. Since PD-L1 largely determines the efficacy and effectiveness of cancer immunotherapies, the development of ATP11B-targeting drugs is highly promising for anti-pancreatic cancer immunity [255].

### 6.10. Sensory systems

The *ATP11A* gene is located within the DFNA33 (Deafness, Autosomal Dominant 33) locus and *ATP11A* SNPs are associated with autosomal dominant nonsyndromic hearing loss [256]. One of the mutations investigated contains an 8-bp duplication which results in normal splicing, but a truncated protein. ATP8B1 has been extensively studied for its role in cholestasis; however, these patients may experience extra-hepatic symptoms, including hearing loss [257]. In mouse models, the *Atp8B1*^*G308V/G308V*^ mutation results in mild cholestasis [214], and these mice experience significant hearing loss [258]. Atp8B1 specifically localizes to stereocilia of the cochlear hair cells, and the reduction of Atp8B1 induces stereocilia degeneration in one-month-old mice [258]. These results suggest that Atp8B1 is necessary to maintain the integrity of the cochlear hair cells. In addition, ATP8A2-deficient mice display shortened photoreceptor outer segments, a substantial reduction in both visual and auditory responsiveness [198].

### 6.11. Skeletal muscle

Regulated exposure of PS on the cell surface of myoblasts is required for cell-cell fusion during myotube formation. Myoblast fusion normally occurs in an end-to-end pattern but ATP11A deficiency in C2C12 myoblasts results in multi-directional fused muscle fibers to produce syncytia rather than myotubes [52]. The ATP11A deficient C2C12 cells had reduced calcium influx which suggested that loss of PS asymmetry inhibits PIEZO1, a calcium ion channel essential for myotube fusion events. Indeed, PIEZO1 activity was recovered when cell surface exposed PS was exchanged for PC. This finding suggests that PS flipping by ATP11A is necessary for PIEZO1 activation, and to prevent unregulated myotube fusion.

### 6.12. Placenta

Trophoblasts fuse together to form multinuclear syncytiotrophoblasts during the formation of the placental labyrinth, where oxygen, nutrients and waste are exchanged between maternal and fetal blood. Homozygous loss of *Atp11A* results in embryonic lethality of mice attributed to a defect in the placental development [259]. The placental labyrinthine layer of *Atp11A*^−/−^ embryos was thinner and had reduced blood vessels than control embryos. The trophoblasts which enable embryo implantation were apoptotic and failed to form a functional syncytium in the *Atp11A* knockout mice. These results suggest that a failure to properly regulate PS exposure and its subsequent return to the cytosolic leaflet disrupts trophoblast fusion leading to placental defects.

## 7. Conclusions and future directions

This review has covered the evolutionary transition from cation pumps to lipid flippases, new structural insights that help us elucidate the mechanism of how flippases translocate lipid species from the outer leaflet to the inner leaflet and a proposed pathway the lipid substrate travels within the membrane, the role of P4-ATPases in cellular trafficking, and the implications of P4-ATPase deficiency and dysfunction in various diseases in both humans and mice, that can affect almost every organ. Much progress has been made in the P4-ATPase field in the last few years, but there is still a need for additional research to fully elucidate the involvement of flippases in cellular physiology and disease and explore their potential as therapeutic targets.

## Figures and Tables

**Table 1:** P4-ATPases in *Saccharomyces cerevisiae* and their human homologs. TGN-trans-Golgi network;PM-plasmamembrane.

α Subunit	β Subunit	Substrate	Cellular localization	Human homologs
Drs2	Cdc50	PS, PE	TGN, endosome	ATP8A1,2, ATP8B1–4, ATP11A,B,C
Neo1	None	PE, PS	Golgi, endosome	ATP9A, ATP9B
Dnf1	Lem3	GlcCer, PC, PE	PM, Golgi, endosome	ATP10A, ATP10B, ATP10D
Dnf2	Lem3	GlcCer, PC, PE	PM, Golgi, endosome	ATP10A, ATP10B, ATP10D
Dnf3	Crf1 (YNR048W)	PS	TGN, PM	ATP8, ATP11?

## Data Availability

No data was used for the research described in the article.
